# Supplementary material for: Tacrolimus dosing in liver transplant recipients using phenotypic personalized medicine: A phase 2 randomized clinical trial
Source: Nat Commun. 2025 May 16;16:4558. doi: 10.1038/s41467-025-59739-6 (PMC12084539; doi:10.1038/s41467-025-59739-6)
Supplement: Supplementary file 1 — Supplementary Information [file 41467_2025_59739_MOESM1_ESM.pdf]

Tacrolimus Dosing in Liver Transplant Recipients Using Phenotypic Personalized Medicine: A Phase 2 Randomized Clinical Trial

Supplementary Information

**Table of Contents**	**Page**
Supplementary Table 1	2
Supplementary Table 2	3
Supplementary Table 3	4
Supplementary Note CONSORT Checklist	5
University of Florida IRB-01 Protocol	7

	Standard of Care	PPM	P-value
	(n=31)	(n=31)	
Male	17 (55%)	18 (58%)	1.0
Female	14 (45%)	13 (42%)	
HCC	3 (10%)	8 (26%)	0.18
SLKT	6 (19%)	3 (10%)	0.47
Redo OLT	1 (3%)	2 (6%)	1.0
Recipient Race/Ethnicity			0.30
Non-Hispanic White	27 (87%)	26 (84%)	
Hispanic White	2 (6%)	4 (13%)	
Non-Hispanic Black	2 (6%)	0	
Asian	0	1 (3%)	
Recipient Age	57 (42-61)	58 (50-63)	0.62
BMI (kg/m^2)	27.7 (24.9-32)	28.6 (22.7-32.6)	0.99
NaMELD	27 (19-29)	27 (15-32)	0.66
Warm Ischemia Time (min)	32 (27-37)	33 (26.5-37.5)	0.78
Cold Ischemia Time (min)	390 (314-440)	333 (301.5-423.5)	0.49
DCD Donor	0 (0%)	1 (4%)	0.49
Donor Age (years)	37 (25-57)	39 (32-54)	0.89
Hepatitis C Positive Donor	2 (6%)	2 (6%)	1.0
Donor Risk Index	1.38 (1.22-1.73)	1.35 (1.18-1.66)	0.45
Dialysis After Transplant	4 (13%)	2 (6%)	0.67
Donor Cause of Death			
Anoxia	9 (29%)	11 (38%)	0.44
Cerebrovascular Accident	11 (35%)	12 (41%)	
Trauma	11 (35%)	6 (21%)	
Fluconazole Use After Transplant	19 (61%)	16 (55%)	0.79
Mycophenolic Acid Use	2 (6%)	2 (6%)	1.0
Basiliximab Use	11 (35%)	8 (25.8%)	0.58
Tacrolimus Target Range Other Than 8-10 ng/mL	4 (13%)	5 (16%)	0.73

Supplementary Table 1. Baseline characteristics of the *randomized* population. HCC: Hepatocellular Carcinoma; SLKT: Simultaneous Liver-Kidney Transplant; OLT: Orthotropic Liver Transplant; BMI: Body Mass Index; NaMELD: Sodium-Model for End-stage Liver Disease. Categorical variables were compared between the two randomized groups using the chi-square test of independence, or Fisher's exact test when expected cell counts were less than five, with statistical significance assessed using a two-tailed test.

	LT				SLKT			
	Both (n=48)	SOC (n=24)	PPM (n=24)	P-value	Both (n=8)	SOC (n=5)	PPM (n=3)	P-value
LOS (d)	15.7 (12.0)	18.0 (11.6)	13.4 (12.2)	0.011	14.4 (4.7)	16.6 (4.2)	10.7 (3.1)	0.07
% large deviation	31 (25)	41 (28)	22 (19)	0.014	33 (21)	26 (25)	43 (9)	0.3

Supplementary Table 2. Comparison of Length of Stay and Percentage of Days with Large Deviations in Tacrolimus Levels Between Liver Transplant (LT) and Simultaneous Liver-Kidney Transplant (SLKT) Recipients. Mean (SD). Categorical variables were compared between the two randomized groups using the chi-square test of independence, or Fisher's exact test when expected cell counts were less than five, with statistical significance assessed using a two-tailed test.

	No HCC				HCC			
	Both (n=46)	SOC (n=26)	PPM (n=20)	P-value	Both (n=10)	SOC (n=3)	PPM (n=7)	P-value
LOS (d)	15.9 (12.0)	17.8 (10.9)	13.2 (11.8)	0.18	14.9 (5)	17.3 (9.7)	12.9 (11.6)	0.56
% large deviation	31 (25)	38.3 (27.3)	26.0 (17.4)	0.068	33 (21)	38.9 (34.7)	19.5 (24.4)	0.45

Supplementary Table 3. Comparison of Length of Stay and Percentage of Days with Large Deviations in Tacrolimus Levels Between Recipients with Hepatocellular Carcinoma (HCC) and Those Without. Mean (SD). Categorical variables were compared between the two randomized groups using the chi-square test of independence, or Fisher's exact test when expected cell counts were less than five, with statistical significance assessed using a two-tailed test.

CONSORT 2010 checklist of information to include when reporting a randomised trial*

Section/Topic	Item No	Checklist item	Reported on page No
**Title and abstract**			
	1a	Identification as a randomised trial in the title	1
	1b	Structured summary of trial design, methods, results, and conclusions (for specific guidance see CONSORT for abstracts)	2
**Introduction**			
Background and objectives	2a	Scientific background and explanation of rationale	4-6
	2b	Specific objectives or hypotheses	6
**Methods**			
Trial design	3a	Description of trial design (such as parallel, factorial) including allocation ratio	17-21
	3b	Important changes to methods after trial commencement (such as eligibility criteria), with reasons	N/A
Participants	4a	Eligibility criteria for participants	18
	4b	Settings and locations where the data were collected	18
Interventions	5	The interventions for each group with sufficient details to allow replication, including how and when they were actually administered	18
Outcomes	6a	Completely defined pre-specified primary and secondary outcome measures, including how and when they were assessed	18-20
	6b	Any changes to trial outcomes after the trial commenced, with reasons	N/A
Sample size	7a	How sample size was determined	18-19
	7b	When applicable, explanation of any interim analyses and stopping guidelines	N/A
Randomisation:			
Sequence generation	8a	Method used to generate the random allocation sequence	16-17
	8b	Type of randomisation; details of any restriction (such as blocking and block size)	16-17
Allocation concealment mechanism	9	Mechanism used to implement the random allocation sequence (such as sequentially numbered containers), describing any steps taken to conceal the sequence until interventions were assigned	16-17
Implementation	10	Who generated the random allocation sequence, who enrolled participants, and who assigned participants to interventions	16-17
Blinding	11a	If done, who was blinded after assignment to interventions (for example, participants, care providers, those	16-17

		assessing outcomes) and how	
	11b	If relevant, description of the similarity of interventions	
Statistical methods	12a	Statistical methods used to compare groups for primary and secondary outcomes	19-21
	12b	Methods for additional analyses, such as subgroup analyses and adjusted analyses	19-21
**Results**			
Participant flow (a diagram is strongly recommended)	13a	For each group, the numbers of participants who were randomly assigned, received intended treatment, and were analysed for the primary outcome	10-11
	13b	For each group, losses and exclusions after randomisation, together with reasons	10-11
Recruitment	14a	Dates defining the periods of recruitment and follow-up	7
	14b	Why the trial ended or was stopped	7
Baseline data	15	A table showing baseline demographic and clinical characteristics for each group	Table 1
Numbers analysed	16	For each group, number of participants (denominator) included in each analysis and whether the analysis was by original assigned groups	7
Outcomes and estimation	17a	For each primary and secondary outcome, results for each group, and the estimated effect size and its precision (such as 95% confidence interval)	7-9
	17b	For binary outcomes, presentation of both absolute and relative effect sizes is recommended	7-9
Ancillary analyses	18	Results of any other analyses performed, including subgroup analyses and adjusted analyses, distinguishing pre-specified from exploratory	8-9
Harms	19	All important harms or unintended effects in each group (for specific guidance see CONSORT for harms)	11
**Discussion**			
Limitations	20	Trial limitations, addressing sources of potential bias, imprecision, and, if relevant, multiplicity of analyses	16-17
Generalisability	21	Generalisability (external validity, applicability) of the trial findings	16-17
Interpretation	22	Interpretation consistent with results, balancing benefits and harms, and considering other relevant evidence	16-17
**Other information**			
Registration	23	Registration number and name of trial registry	3
Protocol	24	Where the full trial protocol can be accessed, if available	21
Funding	25	Sources of funding and other support (such as supply of drugs), role of funders	24

University of Florida IRB-01 Protocol

TITLE:
Optimizing Immunosuppression Drug Dosing via Phenotypic Precision Medicine

INVESTIGATORS AND STUDY STAFF:
Ali Zarrinpar, Mark Johnson, Danielle McKimmy (Dean Ho and Chih-Ming Ho at UCLA)

ABSTRACT:
The investigators have developed a computational platform to rapidly identify optimal drug and dose combinations from the innumerable possibilities. By testing this technique termed Phenotypic Personalized Medicine (PPM) in a diverse number of experimental systems representing different diseases, they have found that the response of biological systems to drugs can be described by a low order, smooth multidimensional surface. The main consequence of this is that optimal drug combinations can be found in a small number of tests. This input-output relationship is always based on experimental data, not modeling, and it would lead to a straightforward solution for handling human diversity in drug dosing needs, among other clinical problems. The investigators will test the hypothesis that PPM can be developed and validated for clinical use by conducting a prospective clinical trial to compare the feasibility and efficacy of this approach to standard of care provider dosing. This group has previously used PPM-based optimization to find novel drug combinations in in vitro and in vivo models of cancer and infection. In a published first-in-human study, they compared 4 PPM-dosed patients and 4 control (standard of care dosed) patients. They calculated the tacrolimus dosing regimen using the PPM process and showed significant improvement in variability and a trend toward improved efficacy in achieving goal drug blood-levels. For this application, they aim to show in a larger clinical trial, that PPM is more effective than unaided (standard of care) dosing. This will allow the generation of data to justify a multi-center confirmatory study and to explore a wider array of clinical outcomes to optimize.

BACKGROUND:
The introduction of calcineurin inhibitors like tacrolimus has greatly reduced the incidence of acute rejection and has improved graft and patient survival after transplantation. However, these drugs have narrow therapeutic ranges and serious side-effects. Inter- and intra-individual variability in dosing requirements, particularly across different patient populations, necessitates empirical physician-titrated drug administration that frequently results in deviation from target ranges, particularly during the critical post-operative phase. Multiple studies have shown that high drug level variability is associated with poor long-term outcomes, including rejection and graft loss. As such, there is a clear need for personalized medicine to address post-transplant immunosuppression. However, a robust procedure to achieve personalized coadministration of tacrolimus and other post-transplant drugs has thus far not been available.
Post-transplant immunosuppression provides a challenging model to test any precision medicine platform. Previous studies have sought to personalize tacrolimus dosing using genetics, population pharmacokinetics, and other predictive modeling approaches. However, it is difficult to simultaneously account for the substantial degree of inter- and intra-individual variability in treatment regimens. These differences also lead to health disparities that are not solely attributable to access, socioeconomics, or adherence. Tacrolimus, one of the most widely used immunosuppressants and a mainstay of solid organ transplantation, has a narrow therapeutic index and wide pharmacokinetic variability; it is a substrate of cytochrome P450 and P-glycoprotein (also known as MDR1 or ABCB1), both with genetically variable expression levels in the intestine and liver. Clearance of tacrolimus is dependent on liver and kidney function, both of

which can vary tremendously in the post-transplant setting. Furthermore, all post-transplant patients are on multiple interacting medications. A simple pharmacogenetic algorithm will not be able to respond adequately to the variability.

We have developed a powerful platform that utilizes patient clinical data to construct a Parabolic Response Surface (PRS). The PRS is patient-specific and based on individualized values that represent each patient's response to drug treatment. Examples of this response can be tacrolimus blood trough levels or quantitative markers of organ function or injury. PRS reconciles clinical data into a visual map that enables the immediate identification of optimal drug doses needed to bring drug levels to within the desired range. Importantly, because the PRS process does not require a priori knowledge of disease mechanism, it can efficiently prescribe precise and optimized drug doses despite the frequent changes to patient treatment regimens following transplantation that can have a profound effect on drug metabolism.

SPECIFIC AIMS:

Overall Aim: Prospective randomized clinical trial applying PPM to tacrolimus dosing in liver and/or kidney transplant recipients to show improvement in maintaining drug trough levels within the target range.

> Sub-Aim 1: Prospective clinical trial comparing PPM-based dosing with standard of care physician guided dosing in liver and/or kidney transplant recipients to show improvement in time-within-target-range of drug trough levels.
>
> > Measures of drug level management will include i) fraction of days outside of target range, ii) fraction of days with large deviations (>2 ng/mL), and iii) ratio of area-under-the-curve outside of target range to total. Clinical measures in both arms will also be monitored to ensure subject safety and correlate trough level maintenance to clinical outcomes.
>
> Sub-Aim 2: Substudy comparing the pharmacokinetics of patients with high variance in tacrolimus dosing with those with low variance.
>
> > To examine contributors to variance in tacrolimus dosing, we will measure the pharmacokinetics of a subset of patients in this study and correlate the findings with donor/recipient genetics, changes in drug regimens, and clinical factors such as liver and kidney function. We will use the pharmacokinetic data to interpret the performance of PPM and provide additional validation for our trial.

RESEARCH PLAN:

The aim of this project is to use PPM to uncover valuable and previously unknown information pertaining to patient dose requirements and correlate them with patient clinical and other contextual information. Importantly, because PPM is able to determine patient-specific levels of drug synergism and antagonism, this project is also expected to reveal vital patient subpopulation information in terms of how the modulation of multiple medications may impact tacrolimus levels and/or precision-based measures of immunosuppression at a patient-specific level. In addition, any future discovery of quantitative biomarkers as measures of immunosuppression will serve as a gateway towards even more effective personalized and relevant drug dosing.

Aim: Prospective randomized clinical trial applying PPM to tacrolimus dosing in liver and/or kidney transplant recipients to show improvement in maintaining drug trough levels within the target range.

Sub-Aim 1: Prospective clinical trial applying PPM to tacrolimus dosing in liver and/or kidney transplant recipients to show improvement in management of drug trough levels. This sub-aim constitutes the crux of the proposal. Its key objectives are: 1) to ensure the safety of the subjects; 2) to test the performance of PPM in guiding tacrolimus dosing in order to keep patient drug levels

within target range more frequently, lead to fewer days out of range, and result in smaller deviations from the target range than the standard of care.

Adult patients who have undergone deceased donor liver and/or kidney transplantation will be recruited and randomized 1:1 to standard of care physician-guided dosing or PPM-guided dosing. Patients with contraindications to tacrolimus will be excluded. Following transplantation, patients will be started on a standard of care medication regimen including tacrolimus. For the first 72 hours tacrolimus will be dosed per standard of care. This allows enough data points to be gathered to allow PPM prediction. Blood tacrolimus trough levels will be taken daily. For standard of care subjects, a senior clinical pharmacist will determine the subsequent doses in consultation with the attending surgeon on call. For PPM subjects, de-identified data consisting of the daily treatment regimen details including drugs already administered, drugs to be administered, and hemodialysis or any other procedures to be performed will be sent to the PPM team at UCLA. Following analysis, the PPM team will suggest a tacrolimus dose for clinician (attending surgeon on call) approval prior to administration. In the rare instance that the UCLA team cannot arrive at a recommendation, then the subject will receive the standard of care (physician-guided) tacrolimus dose. Patients will remain on the trial until discharge from the hospital at which point they will all revert to standard of care dosing. Outcome measures will include i) fraction of days outside of target range, ii) fraction of days with large (> 2 ng/mL) deviations, and iii) ratio of area-under-the-curve outside of target range to total. Clinical safety and efficacy measures will also be monitored to ensure subject safety and to correlate trough level maintenance to clinical outcomes. These include adverse events such as neurotoxicity [documented seizures, clinically significant tremors, or imaging-confirmed posterior reversible encephalopathy syndrome (PRES)], nephrotoxicity (biopsy proven acute kidney injury or calcineurin toxicity, anuria or oliguria requiring dialysis), biopsy-proven rejection, need for repeat steroid pulse, graft loss, or death.

Statistical Analysis. Comparative analysis of the effectiveness of PPM dosing will be assessed using area-under-the-curve and variance analysis as in sub-aim 1 above. Based on our preliminary results thirty patients per group will provide greater than 90% power (alpha = 0.05) to show a difference in a two-tail test.

Feasibility, Anticipated Challenges and Proposed Solutions. The UF transplant program performs more than 150 adult liver and/or kidney transplant operations per year. This allows us to complete recruitment in less than 6 months. We do not anticipate major challenges; Dr. Zarrinpar has substantial experience working on the clinical validation of PPM. We have already successfully performed a prospective pilot study with the same protocol. Because of this we have an established protocol that is conducive to markedly improving the treatment outcomes of patients that participate in the study.

Sub-Aim 2: Comparing the pharmacokinetics of patients with high variance in tacrolimus dosing with those with low variance.

Studies in kidney transplant recipients have indicated that cytochrome P450 CYP3A5 genotype and hematocrit account for more than 60% of variability in tacrolimus pharmacokinetics. Data in liver transplant patients is less well characterized but the unexplained variability is likely higher. To examine contributors to variance in tacrolimus dosing, we will measure tacrolimus pharmacokinetics in this study and correlate the findings with donor/recipient genetics, changes in drug regimens, and clinical factors such as liver and kidney function. For each subject, at least three blood samples (pre-dose and 2 and 6 hours after the dose) will be collected on study days 2, 7, and 14. We will use these pharmacokinetic data to interpret the performance of PPM predictions and provide additional validation for our trial. Genotyping of each donor and recipient

will be performed for CYP3A5 and P-glycoprotein to look for their contribution to variance in pharmacokinetics. Both one- and two-compartment models will be evaluated.

Statistical analysis. Similar to the previous sub-aim, comparisons between the two groups will performed using either the two-tailed Welch's t-test or Student's t-test. For nonparametric (non-normal) distributions, a two-tailed Levene's test will be used to compare variances, and a two-tailed Wilcoxon Rank Sum test will be used to compare medians. The number of subjects in this study is similar to that in the study by Gerard et al in their approach to building a model for determination of tacrolimus trough blood concentrations.

Data Safety Monitoring Plan. The Principal Investigator (PI) will be responsible for ensuring participants' safety on a daily basis. Given the low risk status of this study with minimal impact on patient care, a Data and Safety Monitoring Board will not be required. Furthermore, each liver/kidney transplant patient is closely followed by a large group consisting of the clinical transplant team (surgeons, hepatologists, pharmacist, nurse coordinators) and the transplant administrative team (transplant program managers and quality officers). One-month, one-year, and three-year outcomes are monitored closely and reporting on patient quality is mandated by the United Network for Organ Sharing (UNOS). Dedicated statisticians in the transplant program monitor real-time outcomes and report them in weekly emails and monthly quality meetings.
A Data Safety Monitoring Board will additionally be appointed. It will include a transplant hepatologist, a transplant nephrologist, and a biostatistician not involved in the study. We will perform an interim analysis comparing the occurrence of i) biopsy-proven rejection episodes, and ii) fraction of days with large (> 2 ng/mL) deviations after ten patients have been randomized into each arm (twenty total subjects). The board will meet after enrollment of 10 patients into each arm (to evaluate the interim analysis) and then every six months thereafter until study completion. The study will be stopped in the event there is a significant unacceptable difference between the two groups. The biostatistical consulting service at the CTSI will assist in monitoring study enrollment, safety signals, and achievement of targets.

Protection of patient medical records: To ensure that patient information is de-identified/anonymized, we will abstract all data (images, lab, clinical data etc.) onto a page or file with only the anonymization codes with no personal identifiers or utilize protocols/programs that will be able to redact the personal identifiers when uploading them as data for study. The code key will only be available to the Project PI and authorized personnel and kept in a secure, locked location. Upon completion of the study, this coded template/anonymization key will be discarded according to regulations. The PI will oversee this task.

POSSIBLE DISCOMFORTS AND RISKS:

Risks of PPM-guided dosing of therapeutic agents to optimize patient-specific immunosuppression include:

- The administration of a drug dose that varies from those given based on clinical practice. This may result in over-dosing or under-dosing. The risks of chronic over-dosing include altered mental status and renal injury. The risks of chronic under-dosing include possible rejection. Both of these risks are mitigated by the fact that blood drug levels are tested daily and deviations will be corrected.
- For Sub-Aim 2, additional blood draws will need to be performed (2 and 6 hours after the dose), on postoperative days 2, 7, and 14. (The pre-dose blood draw is standard of care.)

- Patient medical records will need to be accessed during the study. Therefore, there is a risk of exposure of PHI. Patient-record access and subsequent anonymizing and de-identification of records will be implemented (as indicated above).

Protection Against Risks: The PPM process is not automated. PPM-suggested doses are always based on implicit limits set by the clinical team so that doses will always be within clinically-relevant levels. Most importantly, the administered dose will always be selected by the supervising physician following consultation with the PPM team.

The blood draws will be performed by trained phlebotomists and will not exceed 7 mL per blood draw for a total of 42 mL over two weeks at maximum.

POSSIBLE BENEFITS:

The subjects themselves may or may not benefit from being part of this study. The PPM process has the potential to optimize tacrolimus dosing for the patients on the treatment arm. For the population at large, the ability to definitively optimize dosing will enable the accurate correlation of genomic factors with dosing trends. This may ultimately help eliminate health disparities in not only transplantation but other indications as PPM is broadly applicable and scalable.

Importance of Knowledge to Be Gained: While it is well known that dosing trends vary between subpopulations based on demographic or pharmacogenomic profiles, it has been challenging to definitively construct accurate and robust subpopulation-specific regimens due to the previous inability to correlate genomic or ethnic information with dose response. The application of PPM that will result in individualized and optimized regimens to be developed for each patient in this study will allow the investigators to correlate information in a way that was previously not possible. This will open new doors to improving treatment outcomes in the form of graft/patient survival for transplant immunosuppression, or other indications in oncology, infectious diseases, and other applications.

CONFLICT OF INTEREST:

None.
