# Supplementary material for: Tacrolimus dosing in liver transplant recipients using phenotypic personalized medicine: A phase 2 randomized clinical trial
Source: Nat Commun. 2025 May 16;16:4558. doi: 10.1038/s41467-025-59739-6 (PMC12084539; doi:10.1038/s41467-025-59739-6)
Supplement: Supplementary file 3 — Reporting Summary [file 41467_2025_59739_MOESM3_ESM.pdf]

nature portfolio

Corresponding author(s): Ali Zarrinpar

Last updated by author(s): 03/15/2025

Reporting Summary

Nature Portfolio wishes to improve the reproducibility of the work that we publish. This form provides structure for consistency and transparency in reporting. For further information on Nature Portfolio policies, see our Editorial Policies and the Editorial Policy Checklist.

Statistics

For all statistical analyses, confirm that the following items are present in the figure legend, table legend, main text, or Methods section.

n/a	Confirmed	
☐	☒	The exact sample size (*n*) for each experimental group/condition, given as a discrete number and unit of measurement
☒	☐	A statement on whether measurements were taken from distinct samples or whether the same sample was measured repeatedly
☐	☒	The statistical test(s) used AND whether they are one- or two-sided<br>*Only common tests should be described solely by name; describe more complex techniques in the Methods section.*
☐	☒	A description of all covariates tested
☐	☒	A description of any assumptions or corrections, such as tests of normality and adjustment for multiple comparisons
☐	☒	A full description of the statistical parameters including central tendency (e.g. means) or other basic estimates (e.g. regression coefficient) AND variation (e.g. standard deviation) or associated estimates of uncertainty (e.g. confidence intervals)
☐	☒	For null hypothesis testing, the test statistic (e.g. *F*, *t*, *r*) with confidence intervals, effect sizes, degrees of freedom and *P* value noted<br>*Give P values as exact values whenever suitable.*
☒	☐	For Bayesian analysis, information on the choice of priors and Markov chain Monte Carlo settings
☒	☐	For hierarchical and complex designs, identification of the appropriate level for tests and full reporting of outcomes
☐	☒	Estimates of effect sizes (e.g. Cohen's *d*, Pearson's *r*), indicating how they were calculated

Our web collection on statistics for biologists contains articles on many of the points above.

Software and code

Policy information about availability of computer code

Data collection	Data were collected on Microsoft Excel 2016
Data analysis	Statistical analyses were performed on JMP Pro 16.1.0 and confirmed with R 3.5.1.

For manuscripts utilizing custom algorithms or software that are central to the research but not yet described in published literature, software must be made available to editors and reviewers. We strongly encourage code deposition in a community repository (e.g. GitHub). See the Nature Portfolio guidelines for submitting code & software for further information.

Data

Policy information about availability of data

All manuscripts must include a data availability statement. This statement should provide the following information, where applicable:

- Accession codes, unique identifiers, or web links for publicly available datasets
- A description of any restrictions on data availability
- For clinical datasets or third party data, please ensure that the statement adheres to our policy

Deidentified individual participant data, including data dictionaries, will be made available upon publication. Specifically, this includes deidentified participant-level data underlying the results reported in this article, the complete study protocol, statistical analysis plan, and informed consent forms. Source data files for all graphs presented in the figures are included with the publication. These data and documents will become accessible immediately upon article publication and will remain available for a period of five years thereafter. Data access will require approval of a research proposal outlining the intended purpose and scope of analysis, along

with a signed data access agreement. Researchers interested in accessing these materials should contact Ali Zarrinpar at ali.zarrinpar@surgery.ufl.edu. Requests will be reviewed based on scientific merit, relevance of the proposed analysis, and compliance with ethical guidelines. Access will typically be granted for analyses aimed at verifying reported findings or conducting further scientifically relevant research.

Research involving human participants, their data, or biological material

Policy information about studies with human participants or human data. See also policy information about sex, gender (identity/presentation), and sexual orientation and race, ethnicity and racism.

Reporting on sex and gender	Use of Terms:<br>In this study, the terms "sex" and "gender" are used carefully to avoid confusion. "Sex" refers to biological differences, while "gender" refers to roles, behaviors, and identities. In this study population, there was no discordance between sex and gender.<br><br>Findings Application:<br>The findings apply to both male and female patients. Out of the 56 patients included in the final analysis, 32 (57%) were male and 24 (43%) were female.<br><br>Study Design Consideration:<br>Sex and gender were considered in the study design. The trial included all adult participants who underwent liver transplantation; they were randomized into either the SOC or PPM groups.<br><br>Determination Methods:<br>Sex and gender were determined based on self-reported information.<br><br>Source Data:<br>Disaggregated sex data are provided in the study. Out of 56 patients, the baseline characteristics such as the distribution of sex (male and female) and other demographics were recorded and analyzed.<br><br>Overall Numbers:<br>Total Participants: 62 (before exclusions)<br>Final Analyzed Participants: 56<br>Male: 32 (57%)<br>Female: 24 (43%)<br><br>Analyses:<br>Sex-based analyses were performed where applicable. The study reports on the distribution of sex in the randomized groups. No sex or gender-specific analyses were performed given the distribution and number of the subject population.<br><br>Justification for Absence:<br>The lack of such analysis is due to the primary focus being on the overall efficacy of an individualized dosing protocol using PPM versus SOC in tacrolimus dosing, rather than on sex- or gender-specific outcomes. The study design aimed to address inter-individual variability rather than sex- or gender-specific responses. Furthermore, it was not powered to assess differences in sex or gender.
Reporting on race, ethnicity, or other socially relevant groupings	Socially Constructed or Socially Relevant Categorization Variables:<br>The manuscript uses race and ethnicity as socially constructed or socially relevant categorization variables. These variables were included to describe the patient population and to analyze any potential differences in tacrolimus dosing and outcomes that might correlate with these social groupings.<br><br>Reasons for Use:<br>Race and ethnicity were included to provide an adequate description of the patient population and to ensure that the study considered and reported any disparities or differences in clinical outcomes among different social groups. This inclusion aimed to enhance the understanding of how PPM might impact diverse populations and to address potential health disparities. Additionally, this study was funded by the National Institutes of Health (NIH), which mandates the collection of race and ethnicity information in clinical research to ensure comprehensive and inclusive scientific inquiry.<br><br>Definitions and Provision of Terms:<br><br>Race: Categorized as Non-Hispanic White, Hispanic White, Non-Hispanic Black, and Asian.<br>Ethnicity: Categorized primarily by Hispanic or Non-Hispanic status.<br>These categories were self-reported by participants during the enrollment process. The definitions align with common demographic categorizations used in clinical research to facilitate comparison and reproducibility.<br><br>Methods of Classification:<br>Participants provided their race and ethnicity information through self-reporting during the initial screening and enrollment process. The classification relied on participants' identification with specific categories rather than external assignment.<br><br>Control for Confounding Variables:<br>To control for confounding variables in the analyses, the study design incorporated randomization and collection of baseline characteristics.<br>Randomization: Participants were randomly assigned to either the SOC or PPM dosing groups to minimize bias and ensure comparable groups.

	Baseline Characteristics: Detailed baseline characteristics, including race, ethnicity, age, BMI, surgical and medical history, were collected and analyzed to confirm no significant differences between groups at the start of the study.
Population characteristics	Covariate-Relevant Population Characteristics:<br><br>Age: The study population consisted of adults with a median age of 58 years, ranging from 48 to 62 years.<br><br>Genotypic Information: No specific genotypic information was included for the participants, focusing instead on phenotypic outcomes related to tacrolimus dosing. The use of genotyping to inform the initial dosing of tacrolimus presents several challenges in the context of liver transplantation, particularly when involving deceased donors. In deceased donor transplantation, genotyping results are not readily available until at least several days after donor-recipient matching. The matching happens within hours of the transplant operation. Genotyping of neither donor nor recipient is yet a standard practice within the immediate post-transplant period. In addition, genotyping the recipient alone would only provide incomplete information given the critical role of the donor liver in drug metabolism.<br>Furthermore, the early period following liver transplantation is marked by significant variability in physiology, especially in liver, kidney, and intestine. These factors have a profound impact on drug levels and their variability during this period complicates the effective use of genotypic data alone for dose determination. Hence, relying on genotype to guide dosing would overlook the critical influences of these dynamic physiological changes.<br><br>Past and Current Diagnosis:<br>Participants had various underlying conditions necessitating liver transplantation. The primary etiologies included alcohol-related liver disease, non-alcoholic steatohepatitis (NASH), hepatitis C, and hepatocellular carcinoma (HCC). Some participants had undergone previous liver transplants.<br><br>Treatment Categories:<br>Participants were divided into two treatment categories:<br>Standard-of-Care (SOC) Dosing: Participants received clinician-determined dosing.<br>Phenotypic Personalized Medicine (PPM) Dosing: Participants received PPM-guided tacrolimus dosing.<br><br>Baseline Characteristics:<br>The baseline characteristics of the participants included:<br>Body-Mass Index (BMI): The median BMI was 28 kg/m^2, indicating varied body compositions.<br>Recipient Race/Ethnicity: The study included participants from various racial and ethnic backgrounds, predominantly Non-Hispanic White, with some Hispanic White and Non-Hispanic Black participants.<br>Recipient Age: The age distribution was similar across both treatment groups.<br>NaMELD (Sodium-Model for End-stage Liver Disease) Score: Reflecting the severity of liver disease, with a median score indicating the baseline condition of participants.<br>Liver only or combined liver-kidney transplantation<br>Other perioperative and donor specific characteristics.
Recruitment	Recruitment of Participants:<br>Participants were recruited from a single large academic transplant center. All adults scheduled to undergo primary, redo liver, or simultaneous liver/kidney transplantation at the center were screened for eligibility. Randomization occurred immediately prior to the transplant operation to minimize any bias from the operative course.<br><br>Potential recruitment bias:<br>Single-Center Bias: Conducting the study at a single transplant center may limit the generalizability of the findings to other settings or populations.
Ethics oversight	University of Florida Institutional Review Board

Note that full information on the approval of the study protocol must also be provided in the manuscript.

Field-specific reporting

Please select the one below that is the best fit for your research. If you are not sure, read the appropriate sections before making your selection.

☒ Life sciences ☐ Behavioural & social sciences ☐ Ecological, evolutionary & environmental sciences

For a reference copy of the document with all sections, see nature.com/documents/nr-reporting-summary-flat.pdf

Life sciences study design

All studies must disclose on these points even when the disclosure is negative.

Sample size	The sample size for the study was determined based on the results from the pilot study. The primary endpoint of the study was the percentage of days with large (>2 ng/mL) deviations from the target tacrolimus trough level range during the initial post-transplant hospital stay.<br>A sample size of 30 participants per group was planned to achieve 92% power to reject the null hypothesis of equal means between the SOC and PPM groups.
Data exclusions	Several participants were excluded during the study due to specific incidents:

Aborted Transplant Operation: Two patients in the PPM arm did not receive the intervention because their transplant operations were aborted after randomization.
Tacrolimus Discontinuation Due to Neurotoxicity: Tacrolimus was discontinued for one patient in each arm within the first few days after the transplant due to neurotoxicity. These patients were switched to cyclosporine and had no evaluable tacrolimus trough levels within the study period.
Phlebotomy/Laboratory Error: One patient assigned to PPM dosing did not have a tacrolimus level measured on post-operative day 5 due to a laboratory error. This patient was excluded as it would affect dosing accuracy and study outcome measures.
Refusal of Doses: One patient assigned to SOC refused multiple tacrolimus doses in the early post-operative period and was therefore excluded from the study.
Impact of Exclusions:
These exclusions were necessary to ensure the integrity and validity of the study results. By excluding participants who did not meet the criteria or who had significant deviations in treatment, the study aimed to maintain a homogeneous sample and reduce potential confounding factors.

Replication

Measures for Reproducibility:

Pilot Study Validation:
The reproducibility of the findings was initially supported by a pilot study, which demonstrated the feasibility and preliminary efficacy of the PPM approach in tacrolimus dosing. The main study was designed based on these initial results, ensuring a structured and evidence-based methodology.

Randomized Clinical Trial Design:
The study employed a randomized clinical trial design, which is the gold standard for clinical research in order to help eliminate selection bias and allow for the comparison of outcomes between the SOC and PPM groups under controlled conditions.

Blinded Participant Assignment:
Participants were blinded to their group assignments, reducing performance bias and ensuring that any observed differences in outcomes were due to the intervention rather than participant expectations or behaviors.

Standardized Protocols:
All patients were treated according to standardized post-transplant care protocols, which included consistent administration of medications, monitoring of tacrolimus levels, and clinical assessments. This standardization minimized variability in care and ensured that differences in outcomes could be attributed to the dosing strategy.

Multicenter Approach Consideration:
Although this study was conducted at a single center, the methodology and protocols were designed to be replicable in other settings. Future studies are planned to be conducted at multiple sites to further validate the findings and ensure generalizability.

Detailed Statistical Analysis:
Comprehensive statistical analyses were performed to ensure that the results are robust and reproducible.

Data Transparency:
The study protocol, statistical analysis plan, and informed consent forms are available upon request from the corresponding author. This transparency allows other researchers to review the methods and replicate the study if desired.

Replication of Pilot Study Findings:
The main study confirmed the findings of the pilot study with a larger, independent, and more heterogeneous cohort at a different center with a different patient population. This replication provided additional evidence that the PPM approach to tacrolimus dosing is effective and reproducible.

Randomization

Allocation:
SOC Dosing Group: Participants received clinician-determined dosing of tacrolimus.
PPM Dosing Group: Participants received PPM-guided dosing of tacrolimus.

Randomization Method:
Permuted Block Randomization: The randomization was carried out using permuted block randomization with a block size of 4. This method ensures that the allocation of participants to each group is balanced throughout the enrollment process.

Sequence Generation:
The randomization sequence was generated by the principal investigator before the start of the study. This pre-planned sequence was designed to minimize bias and ensure an even distribution of participants between the two groups.

Allocation Procedure:
Screening for Eligibility: All adults scheduled to undergo primary or redo liver or simultaneous liver/kidney transplantation were screened for eligibility based on the inclusion and exclusion criteria.
Informed Consent: Eligible participants or their surrogates provided informed consent to participate in the study.
Randomization and Assignment: Immediately prior to the transplant procedure, participants were randomly assigned to either the SOC or PPM group according to the pre-determined randomization sequence.
Implementation: The PI ensured the proper allocation of participants to the respective groups. To minimize bias from the operative course, group assignment was done just before the transplant operation.

Blinding

Investigator Blinding:

Blinding During Data Collection:
Clinical Team: The clinical team responsible for patient care and administering the interventions was not blinded to group allocation. The nature of the interventions (standard-of-care versus PPM-guided dosing) required the clinical team to know which dosing strategy was being

used to manage the patients effectively. As a result, blinding of the clinical team was not feasible.
Participants: Participants were blinded to their group assignments to prevent performance bias and ensure that their knowledge of the group assignment did not influence their behavior or expectations.

Blinding During Data Analysis:
Data Analysts: The investigators responsible for data analysis were blinded to the group assignments during the statistical analysis phase. This blinding was achieved by coding the groups to conceal their identities (A and B) from the analysts. This approach ensured that the data analysis was conducted without bias.

Rationale for Blinding Decisions:
Clinical Team Blinding: Blinding the clinical team was not possible due to the distinct differences in the intervention protocols between the SOC and PPM groups. The clinical team needed to be aware of the dosing strategy to administer the appropriate care and adjust the dosing as required by the study protocol.
Relevance of Blinding: While blinding the clinical team was not feasible, blinding during data analysis was implemented to mitigate potential bias in interpreting the results. This approach ensured that the outcome assessment remained objective and unbiased.

Blinding of Participants:
Blinding participants to their group allocation was relevant to minimize placebo or nocebo effects that could arise from their knowledge of receiving a novel intervention versus standard care. This blinding helped ensure that the observed effects were due to the intervention itself and not influenced by participants' perceptions or expectations.

Reporting for specific materials, systems and methods

We require information from authors about some types of materials, experimental systems and methods used in many studies. Here, indicate whether each material, system or method listed is relevant to your study. If you are not sure if a list item applies to your research, read the appropriate section before selecting a response.

Materials & experimental systems

n/a	Involved in the study
☒	☐ Antibodies
☒	☐ Eukaryotic cell lines
☒	☐ Palaeontology and archaeology
☒	☐ Animals and other organisms
☐	☒ Clinical data
☒	☐ Dual use research of concern
☒	☐ Plants

Methods

n/a	Involved in the study
☒	☐ ChIP-seq
☒	☐ Flow cytometry
☒	☐ MRI-based neuroimaging

Clinical data

Policy information about clinical studies
All manuscripts should comply with the ICMJE guidelines for publication of clinical research and a completed CONSORT checklist must be included with all submissions.

Clinical trial registration
Optimizing Immunosuppression Drug Dosing Via Phenotypic Precision ClinicalTrials.gov (NCT03527238).

Study protocol
Protocol is attached

Data collection
Setting:
The conduct of the study and the data collection for this study took place at a single large academic transplant center, specifically the University of Florida Health in Gainesville, Florida.

Time Period of Recruitment and Data Collection:
Recruitment Period: Participants were recruited between September 1, 2018, and June 4, 2020. During this period, all adults scheduled for liver transplantation at the center were screened for eligibility.
Data Collection Period: Data collection began with the recruitment of the first participant and continued through their post-transplant hospital stay until discharge. The study included a follow-up period of one year to monitor outcomes, but the primary data collection focused on the immediate post-operative phase.

Outcomes
Primary Outcome Measure:
The primary outcome measure was the percentage of post-transplant days with large (>2 ng/mL) deviations from the target tacrolimus trough level (TTL) range during the initial post-transplant hospital stay.

Rationale for Primary Outcome:
Significance: Large deviations in TTL can indicate improper dosing, leading to increased risk of graft rejection (if levels are too low) or toxicity (if levels are too high). Therefore, maintaining TTL within the target range is critical for patient outcomes.
Threshold: The >2 ng/mL deviation threshold was chosen as it represents clinically significant deviations that could correlate with adverse events.
Pilot Study Basis: This primary outcome was determined based on results from the pilot study, which demonstrated a significant difference in this measure. This was used to power the study, ensuring it had sufficient sample size to detect meaningful differences

between the SOC and PPM groups.

Assessment of Primary Outcome:
Measurement: TTL was measured daily just before the morning dose (12 hours after the previous evening dose) using an automated chemiluminescent assay (CMIA) on the ARCHITECT i2000 platform.
Data Recording: The percentage of days with large deviations from the target range was calculated for each participant.
Comparison: The mean percentage of days with large deviations was compared between the SOC and PPM groups.

Secondary Outcome Measures:
- Percent days outside-of-target range:
o Rationale: This measure provides an overall assessment of how often TTL deviates from the target range, regardless of the magnitude of the deviation.
o Assessment: The percentage of post-transplant days where TTL was outside the pre-defined target range was calculated for each participant and compared between the SOC and PPM groups.
- Length of Hospital Stay (LOS):
o Rationale: Shorter LOS can indicate more effective management of post-transplant care and faster recovery.
o Assessment: LOS was calculated from the day of transplantation to the day of discharge. The median LOS was compared between the SOC and PPM groups.
- Area-Under-the-Curve Outside-of-Target-Range (AUC OOR) Per Day:
o Rationale: AUC OOR measures the extent and duration of deviations from the target TTL range, providing a comprehensive assessment of dosing accuracy.
o Assessment: The AUC OOR was calculated as the sum of the daily deviations from the target range divided by the number of study dosed days. Mean AUC OOR values were compared between the groups.
- Incidence of Biopsy-Proven Graft Rejection:
o Rationale: Graft rejection is a critical outcome in liver transplantation, and its incidence directly reflects the effectiveness of immunosuppression.
o Assessment: The number of biopsy-proven graft rejection episodes during the post-transplant hospital stay was recorded and compared between groups.

Plants

Seed stocks	n/a
Novel plant genotypes	n/a
Authentication	n/a
